# Effects of NSAIDs on the risk factors of colorectal cancer: a mini review

**DOI:** 10.1186/s41021-016-0033-0

**Published:** 2016-04-01

**Authors:** Takahiro Hamoya, Gen Fujii, Shingo Miyamoto, Mami Takahashi, Yukari Totsuka, Keiji Wakabayashi, Jiro Toshima, Michihiro Mutoh

**Affiliations:** Epidemiology and Prevention Division, Research Center for Cancer Prevention and Screening, National Cancer Center, 5-1-1 Tsukiji, Chuo-ku Tokyo, 104-0045 Japan; Department of Biological Science and Technology, Tokyo University of Science, 6-3-1 Niijuku, , Katsusika-ku Tokyo, 125-8585 Japan; Division of Carcinogenesis and Cancer Prevention, National Cancer Center Research Institute, 5-1-1 Tsukiji, Chuo-ku Tokyo, 104-0045 Japan; Central Animal Division, National Cancer Center Research Institute, 5-1-1 Tsukiji, Chuo-ku Tokyo, 104-0045 Japan; Graduate Division of Nutritional and Environmental Sciences, University of Shizuoka, 52-1 Yada, Suruga-ku Shizuoka, 422-8526 Japan

**Keywords:** Non-steroidal anti-inflammatory drugs, Colorectal cancer, Risk factors, Chemoprevention

## Abstract

Evidence from epidemiological and experimental studies has shown that non-steroidal anti-inflammatory drugs (NSAIDs) reduce the risk of colorectal cancer (CRC). The function of NSAIDs and the molecular targets for chemopreventive effects on CRC have been extensively studied and their data were reported. However, the relation between NSAIDs and the risk factors of CRC have not been fully elucidated yet. Thus, relations between NSAIDs and the risk factors of CRC, such as overweight and obesity, alcohol, aging, hypertriglyceridemia and smoking, are summarized with our data and with recent reported data in this review.

## Background

Non-steroidal anti-inflammatory drugs (NSAIDs) relieve pain, reduce inflammation, lower fevers and prevent blood from clotting. Thus, NSAIDs are used to treat inflammatory conditions such as arthritis. One of the traditional NSAIDs, aspirin, was used to protect against heart disease. Conversely, NSAIDs can increase the risk of gastrointestinal (GI) bleedings/ulcer and interfere with kidney function. The severity of side effects increases by taking NSAIDs longer.

A large number of epidemiological and experimental studies have shown that NSAIDs reduce the risk of colorectal cancer (CRC). Meta-analysis of randomized trials revealed that use of aspirin for approximately 5 years reduces incidence and mortality due to CRC by 30–40 % after 20 years of follow-up [1].

The effectiveness of NSAIDS may be attributed to their potent inhibition of cyclooxygenase (COX) enzymes because COX-2 expression and prostaglandin (PG) E_2_ synthesis are elevated in CRC. The COXs/PGH synthases have two enzymes, COX-1 and COX-2, and COX is the limiting enzyme of the PG synthesis pathway. The constitutive enzyme COX-1 has low expression in normal human colorectal tissue, whereas the inducible enzyme COX-2 is elevated in tissue involved in inflammation and in cancer. Traditional NSAIDs block the actions of both COX-1 and COX-2, and selective COX-2 inhibitors are a special category of NSAIDs. In addition, aspirin can inhibit proliferation and induce apoptosis of colon cancer cells [2]. The inhibition and induction by aspirin include the following: (i) the interruption of nuclear factor kappa B (NF-κB), (ii) the interruption of extracellular signal-regulated kinases, (iii) the induction of caspase 8 and 9, and (iv) the inhibition of β-catenin signaling.

As shown above, the function and molecular targets of NSAIDs have been well studied, and several pieces of evidence have shown their chemopreventive effects on CRC. However, the relation between NSAIDs and the risk factors of CRC have not been well examined. In this review, such relationships are summarized with our data and recent reported data in the text, Table 1 and 2.

**Table 1:** Effects of NSAIDs on risk factors of CRC

Risk factors of CRC	NSAIDs effects on each factors
Obesity	Lose weight and reduce the BMI
Alcohol	Increasing risk of GI bleeding due to alcohol
Aging	Slowing
Hypertriglyceridemia	Improvement
Smoking	ー

*BMI* body mass index, *CRC* colorectal cancer, *GI* gastrointestinal

*NSAIDs* Non-steroidal anti-inflammatory drugs

**Table 2:** Effects of NSAIDs plus each risk factors on CRC risk

Risk factors of CRC	NSAIDs effects on CRC risk
Obesity	Decreasing [14–16]
Alcohol	Protection in a social drinker
Aging	ー
Hypertriglyceridemia	Decreasing [49, 50]
Smoking	Increasing [57–59]

*CRC* colorectal cancer, *NSAIDs* Non-steroidal anti-inflammatory drugs

### Risk factors of colorectal cancer

A risk factor is any attribute, characteristic or exposure of an individual that increases the likelihood of developing a disease or injury [3]. In the case of cancer, it is defined as factors that increase the chance of developing cancer. Some people with several risk factors never develop cancer. There is an intensity of correlation between risk factors and cancer. Thus, there might be some weak risk factors of which we are not aware.

Several risk factors of CRC have been demonstrated to date. The World Health Organization (WHO) lists factors that are convincing evidence for the risk of CRC in daily life as follows: (i) consuming red meat, (ii) consuming processed meat, (iii) alcoholic drinks (men), (iv) body fatness / abdominal fatness, and (v) adult attained height increase [4]. Other risk factors are reported as aging [5] and a family history of CRC. Several medical histories of diseases are known to increase the risk of CRC, such as (i) genetic predisposition including familial adenomatous polyposis and hereditary nonpolyposis CRC, (ii) high-risk adenoma, (iii) inflammatory bowel disease, (iv) type-2 diabetes, and (v) hypertriglyceridemia [6]. Moreover, smoking is a strong factor in the development of colorectal adenoma [7].

From recent published literature, we took particular note of overweight and obesity, alcohol, aging, hypertriglyceridemia and smoking. The relation between each risk factor and NSAIDs is described below.

### Obesity and NSAIDs

Obesity is an important risk factor for CRC [8], and there is a significant positive correlation between body mass index (BMI) and CRC risk [9, 10]. Visceral abdominal fat area measured by CT scan is more significantly associated with colorectal adenoma [11]. Visceral fat accumulation causes increases in adipokines such as leptin, inflammatory cytokines such as tumor necrosis factor (TNF) α and interleukin (IL)-6, and growth factors such as insulin-like growth factor (IGF)-I and vascular endothelial growth factor (VEGF) [12, 13]. These factors cause chronic low-grade inflammatory states, increased proliferation and angiogenesis, and promote colon carcinogenesis [12, 13].

The use of NSAIDs is associated with reduced risk of inflammation-associated cancers, and obesity-related cancers are included in inflammation-related cancers [14]. Compared with non-users of NSAIDs, lower risk of obesity-related cancers in NSAID users (hazard ratio (HR) = 0.88; 95 % confidence interval (CI), 0.85–0.92) has been reported [14]. Although the preventive effects of aspirin and NSAIDs on obesity-related cancers are inconclusive, there are several reports showing that aspirin reduced risks of obesity-related cancers including CRC, particularly among obese people. In the Aspirin/Folate Polyp Prevention Study, a randomized controlled trial of aspirin and folic acid to prevent colorectal adenoma, a daily dose of 325 mg aspirin reduced the risk ratio for advanced adenomas compared with placebo among obese subjects (relative risk (RR) = 0.44; 95 % CI, 0.17–1.10) but not among those with normal weight (RR = 1.23; 95 % CI, 0.55–2.77) [15]. In the CAPP2 study that recruited participants with Lynch syndrome, CRC risk was 2.41x greater for obese participants (95 % CI, 1.06–2.96; *P* = 0.03) than for underweight and normal-weight participants, and the obesity-related excess risk of CRC was confined to those randomly assigned to the aspirin placebo group (adjusted HR = 2.75; 95 % CI, 1.12–6.79, *P* = 0.03), but the risk was abrogated in those taking aspirin [16].

PGE_2_ levels in rectal mucosa have been reported to be positively associated with BMI [17]. PGE_2_ has been shown to promote colorectal carcinogenesis [18] and metastasis [19] in mouse models, and inhibition of PGE_2_ synthesis is a potential target for prevention of CRC [20]. Therefore, decrease of obesity-related CRC by NSAID use could be due to inhibition of PGE_2_ production.

Obesity causes insulin resistance with hyperglycemia and hyperinsulinemia, and these contribute to tumor development [21]. Metformin, an insulin resistance-improving, anti-diabetic agent, has been reported to reduce risks of various cancers including colon cancer in diabetic and non-diabetic populations [22, 23]. Metformin inhibits cell proliferation via activation of AMP-activated protein kinase (AMPK) [24]. Serum adiponectin is decreased in obese people, and also activates AMPK and inhibits cell proliferation of colon cancer cells [24]. Recently, it has been reported that aspirin activates AMPK and inhibits mTOR signaling in colon cancer cells [25]. Not all, but several NSAIDs, such as salicylic acid, ibuprofen or diclofenac, which have acidic structures, also induce AMPK activation [26, 27]. This effect is considered to be a COX-independent anti-inflammatory property of aspirin and a subgroup of NSAIDs and may contribute to decrease obesity-related cancer risks.

### Alcohol and NSAIDs

In a meta-analysis of cohort and case–control studies, there are reports describing moderately increased risks of CRC with a dose–response relation for rising alcohol consumption. A polled analysis of eight cohort studies also recorded a dose–response relation between the risk of CRC and the amount of alcohol consumption [28].

The mechanisms by which alcohol intake exerts its carcinogenic effect are not fully understood yet. Acetaldehyde, a metabolite of alcohol, is implicated in esophageal carcinogenesis but is not strongly implicated in colorectal carcinogenesis. Recently, single nucleotide polymorphisms (SNPs) in the alcohol dehydrogenase, *ADH1B,* and aldehyde dehydrogenase, *ALDH2,* of moderate/heavy drinkers were shown to be contributing factors for CRC [29]. Aspirin and salicylate could inhibit both human ADH (metabolize ethanol to acetaldehyde) and ALDH (degradation of acetaldehyde) activities [30]. To date, the effect of aspirin on acetaldehyde production is not fully understood yet.

Ethanol is known as an irritant for the digestive tract. Ethanol intake is known to be an independent risk factor for GI bleeding. It is posited that the GI bleeding risk from aspirin is high in individuals who consume three or more alcoholic drinks every day [31].

Several reports suggest that ethanol exposure alters the cytokine levels and inflammatory status in a variety of tissues in vitro and in vivo, including the colon [32, 33]. Furthermore, chronic alcohol intake promoted intestinal tumorigenesis and tumor invasion in *Apc*^Min/+^ mice [34]. In a report, Wimberly et al. suggested that mast cell-mediated inflammation could be one of the mechanisms by which alcohol promotes intestinal carcinogenesis.

Recently, Landi and colleagues [35] studied the association between SNPs in the IL-6, IL-8, TNFα and PPARG genes and the risk of CRC by a hospital-based case control study. These genes are known to play important roles in inflammation of the colorectum, and common allelic variants are related to changes in biological functions in the inflammation pathway. In their study, Landi and colleagues observed an association between increased CRC risk and the C-allele of a SNP −174 G > C in the IL-6 gene (Odds ratio (OR) = 1.65, 95 % CI, 0.99–2.74). Several reports showed the association of C-allele carriers with inflammatory-related conditions, such as increased plasma levels of C-reactive protein [36], higher serum levels of IL-6 after coronary artery bypass surgery [37] and asymptomatic carotid artery atherosclerosis [38]. In terms of CRC risk, the effect of alcohol drinking was evident only in the subgroup of IL-6 C-allele carriers (OR = 2.19, 95 % CI, 1.3–3.7), and the use of NSAIDs halved the risk from 2.02 (95 % CI, 1.38–2.95) to 1.02 (95 % CI, 0.65–1.61) in the carriers of the C-allele.

These reports suggest that the carcinogenic effect of alcohol may be partly through the induction of acetaldehyde and inflammation, and NSAID use may effectively protect CRC development in a social drinker.

### Aging and NSAIDs

Disruption of normal tissue function dramatically accelerates in old age. Aging is the greatest risk factor for numerous pathologies, including cancer, stroke, neurodegenerative disorders, heart disease and type-2 diabetes [5]. Chronic inflammation is one of the main processes that contributes to age-related disease and causes disruption of normal functioning of tissues. Notably, there is a robust increase of mRNA and secretion of numerous cytokines, chemokines, growth factors and proteases in the senescent cells, and these cells may cause a low level of chronic inflammation systemically during aging [39].

Yeast, nematodes and flies have been recognized as excellent model systems for studying the underlying mechanism of aging and identifying chemicals altering longevity, mainly because of their short lifespans. A growing number of reports showed the effects of NSAIDs on the lifespan extension in yeast [40], nematodes [40, 41], flies [40, 42] and mice [43]. He et al. [40] reported that ibuprofen extended the replicative life span of *Saccharomyces cerevisiae* cells by destabilizing the high-affinity tryptophan transporter. He et al. also found that ibuprofen caused small size at birth and moderate delay in initiation of cell division, which was observed in most long-lived yeast mutants. Meanwhile, celecoxib extended both mean and maximum lifespans in *C. elegans* [41]. The physical health, as indicated by the age-associated decay rate of motor activity, was also significantly improved in celecoxib-treated nematodes without affecting the nutritional value. However, no homologs of mammalian COXs have been identified in unicellular organisms, including *C. elegans*. Furthermore, as analogs of celecoxib that lacked COX-2 inhibitory activity also exhibited a similar effect on nematode lifespan, the anti-aging effect of celecoxib might be independent of its COX-2 inhibitory activity. Indeed, celecoxib was shown to inhibit the activity of 3’-phosphoinositide-dependent kinase-1 (PDK-1), a key component of the insulin/IGF-1 signaling cascade that is involved in lifespan regulation in *C. elegans*. Other studies have demonstrated that NSAIDs have antioxidative effects through anti-radical activity and membrane-stabilizing action [44, 45].

Thus, NSAIDs might be effective for slowing aging and prevention of age-related diseases through not only their anti-inflammatory effects *via* COX-2 inhibitory action but through potential secondary targets including PDK-1 inhibition and the anti-oxidative effect.

### Triglycerides and NSAIDs

The triglyceride values (TG, Neutral fat value) are one of the three indices characterizing metabolic syndrome. Metabolic syndrome is evoked by an accumulation of abdominal fat, and, as previously mentioned, several obesity-associated cancers [46] may occur.

The serum TG value is correlated with a high-fat diet and is higher in patients in an obese state. When the serum TG value increases, colorectal adenomas (often in such as colon polyps) are more likely to occur. This occurrence has been noted in several epidemiological studies. For example, high TGs in hypertriglyceridemia were associated with colorectal adenoma (OR = 1.5, 95 % CI, 1.1–2.0 for the highest versus the lowest quartile, P _trend_ = 0.03). A stronger association was observed between three or more adenoma cases and study controls (OR = 2.3, 95 % CI, 1.3–4.2, P _trend_ < 0.001) [7]. In Japanese men, risk of CRC is increased under high-TG levels [6]. Animal experiments supported the relation between high-TG and carcinogenesis by explaining the underlying mechanisms. Thus, high TG might be understood as a risk factor for CRC [47, 48].

On the other hand, there are a variety of discussions about whether NSAIDs can adjust the TG value. NSAIDs can inhibit the enzymatic activity of COX and attenuate the expression level of PGE_2_. PGE_2_ is activated through an EP3 receptor at the thermoregulatory center in the hypothalamus and functions to raise the set point of body temperature by increasing the cellular metabolism. This linkage means NSAIDs decrease TG levels. Furthermore, PGE_2_ induces the expression of TNFα, and the TG value also increases because of the inhibition of lipoprotein lipase (LPL) [49]. LPL catalyzes the hydrolysis of plasma TG.

The lowering of the TG value by NSAIDs is also found in several studies in the literature. For example, when NSAIDs were used in animal experimental models, they significantly reduced the total cholesterol, TG and low-density lipoprotein (LDL) concentrations in the plasma of hyperlipidemic rats [49, 50].

Niho et al. investigated the influence of the general COX inhibitor, indomethacin, in Min mice and found that treatment with 10 ppm indomethacin in the diet for 14 weeks caused 90 % reduction in serum TG values, along with a reduction in the number of intestinal polyps to 25 % of the untreated control value [51]. In this experiment, LPL mRNA levels in the liver were slightly increased by indomethacin treatment. In humans, indomethacin does not affect serum lipids.

In another model animal experiment, aspirin could improve serum high-TG. Human apolipoprotein CI (apoCI)-expressing mice (APOC1 mice), an animal model with elevated plasma TG levels, as well as normolipidemic wild-type mice, were fed a high-fat diet and treated with aspirin. Aspirin treatment reduced hepatic NF-κB activity in high-fat diet-fed APOC1 and wild-type mice, and in addition, aspirin decreased plasma TG levels (−32 %, *P* < 0.05) in hypertriglyceridemic APOC1 mice. This TG-lowering effect could not be explained by enhanced VLDL-TG clearance, but aspirin selectively reduced hepatic production of VLDL-TG in both APOC1 (−28 %, P < 0.05) and wild-type mice (−33 %, P < 0.05) without affecting VLDL-apoB production [52]. In humans, higher proportions of patients in the salsalate (one of the NSAIDS) treatment groups experienced decreasing circulating TG values and increasing adiponectin concentrations [53]. For preventing colorectal carcinogenesis, significant consideration should be given to the use of NSAIDs to decrease plasma TG levels.

### Smoking and NSAIDs

Smoking is a strong risk factor for the incidence of colorectal adenomas [7] but a weak risk factor for the colorectal adenocarcinomas [54]. Conversely, NSAIDs including aspirin are known to reduce the adenoma recurrence rate. Evidence has accumulated that shows current smoking abrogates or inversely affects the use of aspirin.

We recently performed two trials using enteric-coated aspirin and found that aspirin similarly increased the risk of colorectal adenomas in current smokers [55]. One of these trials is the J-CAPP Study [56]. It is a randomized controlled trial involving 311 patients from Asia with colorectal adenomas and/or early-stage adenocarcinomas (adenocarcinomas with invasions confined to the mucosa) that evaluated the effects of 100 mg/day enteric-coated aspirin for two years. In subgroup analyses, we found a reduced adenoma recurrence rate with an OR of 0.37 (95 % CI, 0.21–0.68) in non-smokers (never-smokers/ex-smokers) and 3.45 (95 % CI, 1.12–10.64) in current smokers. The other trial is the J-FAPP Study II [57]. It is also a randomized controlled trial. It involved 34 subjects with Asian familial adenomatous polyposis that evaluated the effects of 100 mg/day enteric-coated aspirin for 6 to 10 months. In the trial, the OR for a reduction in the diameter of polyps was 0.10 (95 % CI, 0.01–0.98) in non-smokers and 3.00 (95 % CI, 0.15–59.89) in current smokers.

After our findings, two papers confirmed the effects of aspirin use on the status of smoking for colorectal adenoma recurrence. A randomized, double-blind, placebo-controlled trial was performed at centers in Europe, Russia, or the US using 75 mg aspirin for 3 years for patients with 1 or more sporadic adenomas removed from the colon or rectum [58]. Its subgroup analyses revealed OR values of 0.65 (95 % CI, 0.26 − 1.22) and 1.70 (95 % CI, 0.70–4.09) in non-smokers and current smokers, respectively. The other paper was a cross-sectional study including 2,918 consecutive colonoscopy patients over a 30-month period at a university hospital in the US [59]. The incidental rate ratio (IRR) of polyps was 1.72 (95 % CI, 1.46–2.02) in active smokers and 0.73 (95 % CI, 0.61–0.86) in daily aspirin users compared to those without aspirin. Current smoking interacts significantly with aspirin, resulting in an IRR of 1.69 (95 % CI, 1.28–2.24) that shows loss of aspirin protection.

The mechanism by which smoking influences the effect of aspirin is unknown. One clue is that smoking may be associated with resistance to aspirin, possibly through excessive thromboxane production [60, 61]. Further studies are warranted because this issue is very important for the clinical use of aspirin in the future.

## Conclusion with recent recommendation of low-dose aspirin

On September 15, 2015, the U.S. Preventive Services Task Force (USPSTF) posted a draft recommendation statement with several limitations about taking low-dose aspirin every day to help prevent cardiovascular disease (CVD), such as heart attack and stroke, and CRC [62]. They stated that patients aged 50–59 who have a 10 % or greater 10-year CVD risk [63] but who are not at increased risk for bleeding and have at least a 10-year life expectancy fall within the USPSTF B recommendation to take low-dose aspirin daily for at least 10 years. The definition of the B recommendation is that there is high certainty that the net benefit of low-dose aspirin is moderate, or there is moderate certainty that the net benefit is moderate to substantial [62].

The USPSTF also posted that it is insufficient to assess the benefit and harm of aspirin use for those older than 80, and it recommended the use of aspirin for prevention of stroke in women < 55 and for prevention of myocardial infarction in men < 45. For several NSAIDs, such as diclofenac, the NSAIDs will increase the risk of death and recurrent myocardial infraction in patients with prior myocardial infraction [64]. From our data, smoking status should be included in the USPSTF recommendation statement. Use of NSAIDs for pain relief may increase physical activation in a convincing way to reduce the risk of CRC [4]. It will be worthwhile to know the effects of NSAIDs on the risk factors of CRC and utilize them for a person’s physical health as well as to prevent CRC.

## Abbreviations

ADH: Alcohol dehydrogenases
ALDH: Aldehyde dehydrogenase
AMPK: AMP-activated protein kinase
BMI: Body mass index
CI: Confidence interval
COX: Cyclooxygenase
CRC: Colorectal cancer
CVD: Cardiovascular disease
GI: Gastrointestinal
HR: Hazard ratio
IGF: Insulin-like growth factor
IL: Interleukin
IRR: Incidental rate ratio
LDL: Low-density lipoprotein
LPL: Lipoprotein lipase
NSAIDs: Non-steroidal anti-inflammatory drugs
NF-κB: Nuclear factor-kappaB
PDK-1: 3’-phosphoinositide-dependent kinase-1
PG: Prostaglandin
RR: Relative risk
OR: Odds ratio
SNPs: Single nucleotide polymorphisms
TG: Triglyceride
TNF: Tumor necrosis factor
USPSTF: U.S. Preventive Services Task Force
WHO: World Health Organization
